# Supplementary material for: P-TRAP: a Panicle Trait Phenotyping tool
Source: BMC Plant Biol. 2013 Aug 29;13:122. doi: 10.1186/1471-2229-13-122 (PMC3848748; doi:10.1186/1471-2229-13-122)
Supplement: Additional file 7 — P-TRAP source code. The Netbeans project that contains the source code of the application. [file 1471-2229-13-122-S7.zip › javadoc.zip!/javadoc/org-bioalg-widgetfactory/index-all.html]

Index (WidgetFacory)


JavaScript is disabled on your browser.


- Package
- Class
- Tree
- Deprecated
- Index
- Help

*org.bioalg.widgetfactory 1.0*

- Prev
- Next

- Frames
- No Frames

- All Classes

A B C G I O P S V W 


## A

actionPerformed(ActionEvent) - Method in class org.bioalg.widgetfactory.WidgetPopUpMenuHandler


attachLabel(Scene, String) - Method in class org.bioalg.widgetfactory.CircleWidget
:   Add a label the widget


## B

buidlallcommands() - Method in class org.bioalg.widgetfactory.WidgetPopUpMenuHandler


## C

calculateClientArea() - Method in class org.bioalg.widgetfactory.CircleWidget


calculateClientArea() - Method in class org.bioalg.widgetfactory.ParticleWidget


CircleWidget - Class in org.bioalg.widgetfactory
:   Create a circle widget to use used in the graph editor.

CircleWidget(Scene, double, Color) - Constructor for class org.bioalg.widgetfactory.CircleWidget
:   The constructor function.


## G

getColor() - Method in class org.bioalg.widgetfactory.CircleWidget


getColor() - Method in class org.bioalg.widgetfactory.ParticleWidget


getColorBycommand(WidgetPopUpMenuHandler.WIDGET\_COMMANDS) - Method in class org.bioalg.widgetfactory.WidgetPopUpMenuHandler


getcommandByColor(Color) - Method in class org.bioalg.widgetfactory.WidgetPopUpMenuHandler


getLabelString() - Method in class org.bioalg.widgetfactory.ParticleWidget


getPolygon() - Method in class org.bioalg.widgetfactory.ParticleWidget


getPopupMenu(Widget, Point) - Method in class org.bioalg.widgetfactory.WidgetPopUpMenuHandler


getRadius() - Method in class org.bioalg.widgetfactory.CircleWidget


## I

isfixed() - Method in class org.bioalg.widgetfactory.CircleWidget


isfixed() - Method in class org.bioalg.widgetfactory.ParticleWidget
:   A particle is fixed if it is changed by the user.


## O

org.bioalg.widgetfactory - package org.bioalg.widgetfactory


## P

paintWidget() - Method in class org.bioalg.widgetfactory.CircleWidget


ParticleWidget - Class in org.bioalg.widgetfactory
:   This class manages the dealing with particles in the editor.

ParticleWidget(String, Polygon, Color, Scene) - Constructor for class org.bioalg.widgetfactory.ParticleWidget
:   The constructor function


## S

setColor(Color) - Method in class org.bioalg.widgetfactory.CircleWidget


setColor(Color) - Method in class org.bioalg.widgetfactory.ParticleWidget


setIsfixed(boolean) - Method in class org.bioalg.widgetfactory.CircleWidget


setIsfixed(boolean) - Method in class org.bioalg.widgetfactory.ParticleWidget


setLabelString(String) - Method in class org.bioalg.widgetfactory.ParticleWidget


setPolygon(Polygon) - Method in class org.bioalg.widgetfactory.ParticleWidget


setRadius(double) - Method in class org.bioalg.widgetfactory.CircleWidget


setupCommands(ArrayList<WidgetPopUpMenuHandler.WIDGET\_COMMANDS>) - Method in class org.bioalg.widgetfactory.WidgetPopUpMenuHandler
:   Set up the commands of the menu.

shiftPoints() - Method in class org.bioalg.widgetfactory.ParticleWidget
:   Normalize the contour points to the center of the rectangle of the particle.


## V

valueOf(String) - Static method in enum org.bioalg.widgetfactory.WidgetPopUpMenuHandler.WIDGET\_COMMANDS
:   Returns the enum constant of this type with the specified name.

values() - Static method in enum org.bioalg.widgetfactory.WidgetPopUpMenuHandler.WIDGET\_COMMANDS
:   Returns an array containing the constants of this enum type, in
    the order they are declared.


## W

WidgetPopUpMenuHandler - Class in org.bioalg.widgetfactory
:   This is a general widget's pop up menu handler.

WidgetPopUpMenuHandler(GraphScene, ArrayList<WidgetPopUpMenuHandler.WIDGET\_COMMANDS>) - Constructor for class org.bioalg.widgetfactory.WidgetPopUpMenuHandler


WidgetPopUpMenuHandler(GraphScene) - Constructor for class org.bioalg.widgetfactory.WidgetPopUpMenuHandler


WidgetPopUpMenuHandler.WIDGET\_COMMANDS - Enum in org.bioalg.widgetfactory
:   Command types.

A B C G I O P S V W

- Package
- Class
- Tree
- Deprecated
- Index
- Help

*org.bioalg.widgetfactory 1.0*

- Prev
- Next

- Frames
- No Frames

- All Classes
